# LncRNA H19 as a Competing Endogenous RNA to Regulate AQP Expression in the Intestinal Barrier of IBS-D Patients

**DOI:** 10.3389/fphys.2020.602076

**Published:** 2021-01-12

**Authors:** Guanqun Chao, Zhaojun Wang, Yi Yang, Shuo Zhang

**Affiliations:** ^1^Department of General Practice, Sir Run Run Shaw Hospital, Zhejiang University, Hangzhou, China; ^2^Department of Gastroenterology, The First Affiliated Hospital, Zhejiang Chinese Medical University, Hangzhou, China

**Keywords:** LncRNA H19, IBS-D, AQP1, AQP3, mechanism

## Abstract

**Objective:**

The study aimed to investigate the role of Long non-coding RNA (LncRNA) H19 in the pathogenesis of Diarrhea Irritable Bowel Syndrome (IBS-D), and further to the regulatory effect of LncRNA H19 on AQP1, 3 in the intestinal mucosa of IBS-D patients, so as to seek a new way to elucidate the mechanism of IBS in clinic.

**Methods:**

The levels of LncRNA H19, AQP1, and AQP3 were detected in colonic tissues of IBS-D patients, compared with that in healthy controls. Through RNA gene interference and activation methods, small activating RNA (saRNA) and small interfering (siRNA) were transfered into Caco-2 cells *in vitro* experiment, and sub-group for two control group, siH19 empty vector group, siH19 interference group, overexpression H19 vector group, and overexpression H19 empty vector group. Quantitative real-time reverse transcription-polymerase chain reaction (qRT-PCR) and Western blot were applied to evaluate the expression levels of LncRNA H19 and the amount of AQP1 and AQP3 protein expression, respectively.

**Results:**

Compared with healthy volunteers, the levels of LncRNA H19, AQP1, and AQP3 in the colonic mucosa of IBS-D patients were significantly decreased (*P* < 0.05). The results *in vitro* transfection experiment revealed that the level of LncRNA H19 in the siH19 interference group was significantly declined (*P* < 0.05), while there was a remarkable increase in the overexpression H19 vector group (*P* < 0.05), compared with the corresponding control groups. The expression of AQP1 and AQP3 in Caco-2 cells was of positive correlation with the level of LncRNA H19.

**Conclusion:**

That the down-regulation of LncRNA H19 resulted in the expression changes of AQP1 and AQP3 may play an important role in the occurrence and development of IBS-D.

## Introduction

Irritable bowel syndrome (IBS) is a common chronic functional bowel disease, characterized by no organic lesions, but recurrent abdominal pain, abdominal distension, and (or) changes in stool characteristics (Kesuma et al., 2019). As the most common clinical subtype of IBS, diarrhea-predominant irritable bowel syndrome (IBS-D) is accompanied by mental and emotional abnormalities that affect the living quality of patients, except the shared features (Drossman and Hasler, 2016). The incidence of this disease is higher than other gastrointestinal diseases in developing and developed countries, and it may be even higher in western developed countries (Drossman and Hasler, 2016). But when it comes to the exact pathogenesis, it is not very clear yet. Interestingly, a growing number of scholars have paid close attention to the mechanisms of intestinal permeability in recent years. Animal experiments have confirmed that the intestinal permeability of IBS-D mice was significantly increased (Hou et al., 2019). Our previous studies also demonstrated that IBS-D resulted from the permeability enhancement of intestinal mucosa, which might be related to the lower expression of aquaporin (AQP) 1,3,8 (Chao and Zhang, 2018). Particularly, there was an apparent connection between the decrease of AQP3 and diarrhea symptoms (Camilleri et al., 2019). A study suggested that the down-regulation of AQP3 may damage the integrity of intestinal barrier by opening the tight junction complex, thus causing intestinal barrier dysfunction (Zhang et al., 2011). However, more convincing evidence is supposed to be provided to clarify how AQP3 participates in the pathogenesis of IBS-D, and what are the regulatory pathways associated with AQP3 leading to intestinal permeability changes.

Long non-coding RNAs (LncRNAs) are a large class of non-protein coding transcripts with more than 200 nucleotides. They function as intracellular signals, decoys, guides, and scaffolds (Geng et al., 2018), playing an important part in many diseases. As a member of this family, LncRNA H19 was confirmed to be involved in the pathogenesis of atherosclerosis, diabetic retinopathy, ischemic stroke, and certain cancers (Pan, 2017; Lei et al., 2018; Thomas et al., 2019; Xiao et al., 2019). Up to now, though there have been no studies directly explored the role of LncRNA H19 in IBS-D, it was reported that elevation of H19 abundance is also involved in regulating the epithelial barrier functions (Leighton et al., 1995; Juan et al., 2000), which determines intestinal permeability. Here, we investigated the impact of H19 on intestinal barrier dysfunction induced by IBS-D, and its role in modulating AQP1, 3 expression.

## Materials and Methods

### Experimental Human Tissue Specimens

IBS-D patients admitted to the Department of Gastroenterology, the First Affiliated Hospital of Zhejiang Chinese Medical University were included, between December 2018 and October 2019. Healthy volunteers were recruited as controls. Specimens of colonic mucous tissues were obtained from 10 IBS-D patients and 10 healthy subjects who had undergone gastroscopy and enteroscopy in the hospital. This study was approved by the Ethics Committee of the First Affiliated Hospital of Zhejiang Chinese Medical University. Every patient provided written informed consent before the clinical trial. The study methods conformed to the standards set by the Declaration of Helsinki.

#### Inclusion Criteria

(1) Meeting the diagnostic criteria of IBS-D in Rome IV (Drossman and Hasler, 2016);
(2) Inspection results of colonoscopy, B-ultrasound, and fecal occult blood were normal;
(3) No anti-allergic drugs and non-steroidal anti-inflammatory drugs in the latest month;
(4) Age ranged from 18 to 70 years old;
(5) Participating in the trial voluntarily and signing informed consent.

#### Exclusion Criteria

(1) Failing to meet the diagnostic criteria of IBS-D in Rome IV;
(2) Patients with a history of inflammatory bowel disease, digestive tract tumor, and other digestive system diseases;
(3) Patients with serious diseases of cardiovascular, respiratory, urinary, blood, or nervous system;
(4) Pregnant or lactating women;
(5) Symptomatic patients with an acute intestinal infection;
(6) Patients with allergic diseases such as asthma.

### AQP1 and AQP3 Were Detected by Immunohistochemistry

Colonic tissue blocks taken from the ileocecal region about 4–6 cm away from the distal ileocecal valve were washed and dehydrated in ascending series of ethanol, fixed in 4% formalin for 24 h and embedded routinely with paraffin, every section was 3–5 μm. Then the two-step EnVision^TM^ detection kit (Dako, Denmark) was used for immunohistochemistry (IHC) according to the manufacturer’s instruction, undergoing the procedures of dewaxing and rehydration, antigen repair, blocking, incubation and rinsing, DAB staining, etc. The brown or yellow positive particles were exclusively localized in the cytoplasm. A quantitative analysis was made by using the Image-Pro Plus 6.0 software (Media Cybernetics, Inc., United States), which depends on the average integrated optical density (IOD/area ratio. The larger the ratio, the stronger the signal intensity.

### The Expression of LncRNA H19 Was Detected by qRT-PCR

Total RNA was extracted from frozen tissues and cells using TRIzol reagent (KeyGen Biotech Co., Ltd., Najing, Jiangsu, China). Reverse transcription using the mRNA reverse transcription kit (KeyGen Biotech Co., Ltd., Nanjing, Jiangsu, China) was performed according to the manufacturer’s instructions. And the reverse transcription sequences were used for detection. qRT-PCR was carried out using a Real-Time PCR System (Bio-RAD Inc. United States). The procedures included thermal deformation (95°C for 10 s), qPCR × 40 cycles (95°C for 10 s and 60°C for 20 s), and dissociation curve (95°C for 60 s, 55°C for 30 s, and 95°C for 30 s) with gene GAPDH as the internal control. We analyzed RNA for expression by RT2-PCR assays, with expression results calculated using 2^–Δ^ ^Δ^ ^*CT*^-based fold-change: ΔΔCT = (ΔCt_*target*_ −ΔCt_*GAPDH*_)_*IBS*–*D group*_ − (ΔCt_*target*_ −ΔCt_*GAPDH*_)_*control group*_. Primer sequences used for qRT-PCR are listed in Table 1.

**TABLE 1:** Primer sequences used in the study for qPCR detection.

Gene name	Primers	Sequences (5′–3′)
Lnc RNA H19	Forward	5′-TACAACCACTGCACTACCTG-3′
	Reverse	5′-TGGAATGCTTGAAGGCTGCT-3′
GAPDH	Forward	5′-GGGAGCCAAAAGGGTCAT-3′
	Reverse	5′-GAGTCCTTCCACGATACCAA-3′

### *In vitro* Cell Experiment

The *in vitro* experiment was divided into six groups: group A for H19 overexpression vector group, group B for H19 empty vector group, group C for overexpression control group; and group D for siH19 interference vector group, Group E for interference empty group, Group F for interference control group.

Caco-2 cells were cultured in the DMEM medium (Cell bank, Chinese Academy of Sciences, Shanghai, China) with 10% fetal bovine serum (FBS), at the condition of 37 degrees Celsius, 5% carbon dioxide and saturated humidity. Lipo8000i transfection reagent-siRNA were mixed according to the Lipo8000^TM^ transfection reagent instruction (Biyuntian Biotechnology Co., Ltd., Shanghai, China). After the mixture was added, plasmid transfection was carried out in a six-well plate. Gene detection or protein determination would be performed after 2 days’ continuous culture. The method of detecting H19 by qRT-PCR is described above. Primer sequences used for qRT-PCR are listed in Table 1.

AQP protein measurements were performed by Western blots. Antibody AQP1 (Santa Cruz Biotechnology, Inc., United States) and antibody AQP3 (Absin, Shanghai, China) were used. The target proteins were obtained after cell lysis and centrifugation. Then the samples were put on the prepared sepharose gel for electrophoresis (Bio-RAD, United States) and later transferred to the PVDF membrane (Millipore, United States) for electric rotation, with the parameter of 200 mA current for 90 min. After being taken out, the membrane was placed into the blocking solution, subsequently incubated with antibodies twice. Finally, the cleaned membrane was placed on the preservative film for titration, chemiluminescence method was used for detection, and images were collected for analysis.

### Statistical Analysis

SPSS23.0 software was used for statistical analysis (SPSS Inc., Chicago, IL, United States) and Graph Prism 6.0 software (GraphPad Prism, San Diego, CA, United States) for all graphs. All data were expressed as mean ± SD. A Student’s *t*-test was used to analyze differences in H19 expression between healthy control and IBS-D patients. One-way analysis of variance (ANOVA) was used for cultured cells comparison. *P* < 0.05 and *P* < 0.01 were considered as statistically significant and very significant.

## Results

### AQP1 and AQP3 Expression in the Intestinal Mucosa of IBS-D Patients

**FIGURE 1:**
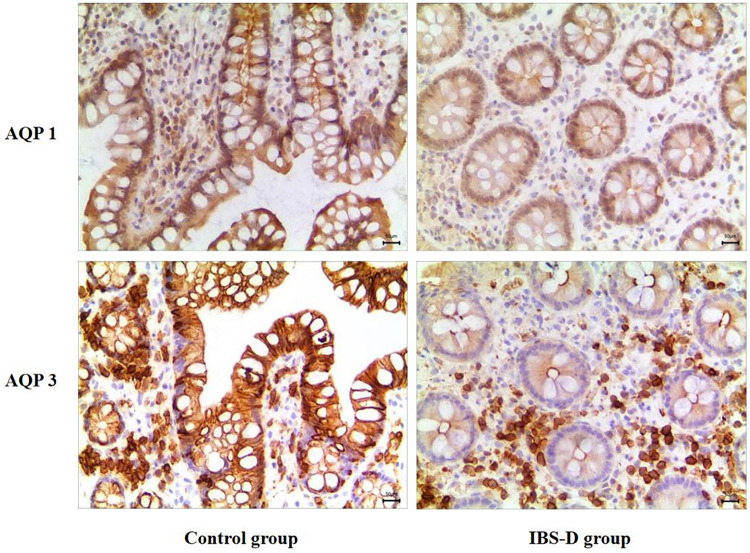
Immunohistochemical results of AQP1 and AQP3 in the two groups of subjects (×200). As shown in the picture, the brown or yellow positive stained particles mainly localized in the cytoplasm of the cells, rather than in the nuclei. Distributed in the cell membrane of intestinal mucosa cells, the amount of AQP1, 3 in patients with IBS-D was less than that in the control group.

As shown in Figure 1, the yellow or brown stained positive particles mainly localized in the cytoplasm of the cells, rather than in the nuclei. It shows that AQP1 and AQP3 were mainly distributed in the cell membrane and cytoplasm of intestinal mucosa cells. And the abundance of the two proteins in IBS-D colonic mucosa was significantly lower than that in the control group (*P* < 0.01) (Table 2).

**TABLE 2:** Expression of AQP1 and AQP3 in two groups of subjects.

Group	AQP1	AQP3
Control group (*N* = 10)	0.340.04	0.330.03
IBS-D group (*N* = 10)	0.220.02*	0.110.01*

The expression of AQP1 and AQP3 in patients with IBS-D and health controls was detected using Ratios of IOD/area, which was performed for immunohistochemical quantity analysis. Correlations among the two groups were analyzed using SPSS23.0 software. AQP proteins expression was performed on biopsy of human. *P < 0.05, vs. control.

### LncRNA H19 Expression in the Colon of IBS-D Patients

**FIGURE 2:**
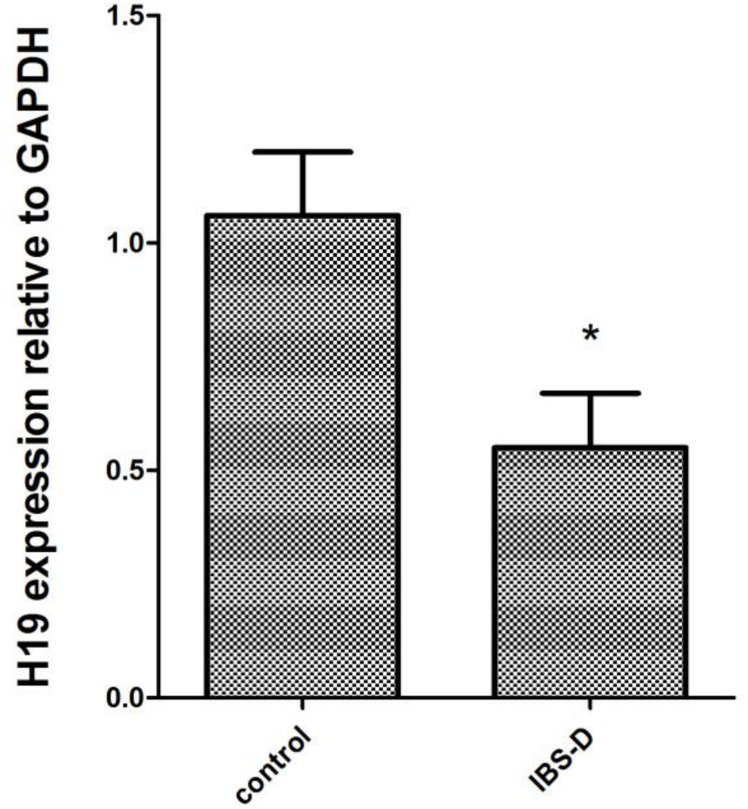
H19 expression in the two groups of subjects by qRT-PCR. The expression of LncRNA H19 in patients with IBS-D and health controls was detected using qRT-PCR based on biopsies. Correlations among the two groups were analyzed by SPSS23.0 software. *P < 0.05, vs. control.

Compared with the control group (1.06 ± 0.14), the relative expression of LncRNA H19 in the IBS-D group (0.55 ± 0.12) was reduced significantly (*P* < 0.05, Figure 2).

### LncRNA H19 Expression *in vitro* Caco-2 Cells Experiment

The results of LncRNA H19 expression in Caco-2 cells after transfection were shown in Table 3. Compared with the overexpression control group (1 ± 0.37) and overexpression empty group (1.34 ± 0.65), the relative expression of LncRNA H19 in the H19 overexpression vector group (83.50 ± 19.90) was increased significantly (*P* < 0.05). In the part of interfering with siH19, compared with the interference control group (1 ± 0.33) and interference empty group (1.35 ± 0.07), the relative expression of LncRNA H19 in the siH19 interference vector group (0.096 ± 0.01) was significantly decreased (*P* < 0.05).

**TABLE 3:** *In vitro* analysis in Caco-2 cells models for H19 transfection and siH19 interference.

Group	Group A	Group B	Group C	Group D	Group E	Group F
H19	83.5019.90*	1.340.65	10.37	0.0960.01*	1.350.07	10.33
AQP1	1.910.03	2.010.06	3.060.14*	1.900.05	1.830.02	1.330.08*
AQP3	4.960.08	5.100.02	11.830.96*	2.440.03	2.400.08	1.480.10*

Expression of LncRNA H19 in Caco-2 cells was detected by qRT-PCR. The amount of proteins AQP1 and AQP3 were measured using western blot. *P < 0.05, vs. corresponding control. Group A: H19 overexpression vector group; Group B: overexpression empty group; Group C: overexpression control group; Group D: siH19 interference vector group; Group E: interference empty group; Group F: interference control group.

### AQP1 and AQP3 Expression *in vitro* Caco-2 Cells Experiment

**FIGURE 3:**
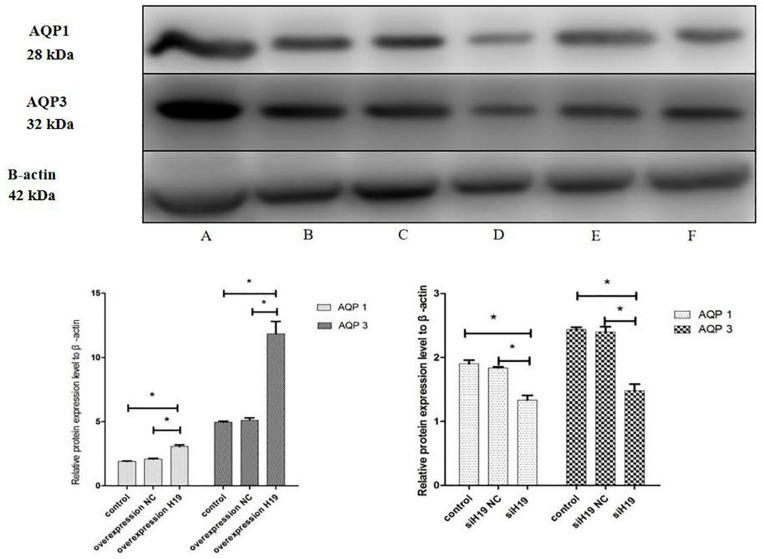
*In vitro* AQP proteins expression detected using Western blot. *In vitro* Caco-2 cells models, levels of AQP1 and AQP3 measured by western blot in group A were higher significantly than that in group B and group C. And protein expression in group D was lower than that in group E and group F. *P < 0.05, vs. corresponding control. Group A: H19 over-expression vector group; Group B: over-expression empty group; Group C: over-expression control group; Group D: siH19 interference vector group; Group E: interference empty group; Group F: interference control group.

Compared with the overexpression control group (1.91 ± 0.03) and the overexpression empty group (2.01 ± 0.06), the relative expression of AQP1 in the H19 overexpression vector group (3.06 ± 0.14) was increased significantly. The relative expression of AQP3 in the H19 overexpression vector group (11.83 ± 0.96) was higher than that in the overexpression empty group (4.96 ± 0.08) and that in the control group (5.10 ± 0.02). Compared with the interference control group (1.90 ± 0.05) and interference empty group (1.83 ± 0.02), the relative expression of AQP1 in the siH19 interference vector group (1.33 ± 0.08) was decreased significantly. The relative expression of AQP3 in the siH19 interference vector group (1.48 ± 0.10) was significantly lower than that in the interference control group (2.44 ± 0.03) and the interference empty group (2.40 ± 0.08) (all *P* < 0.05, Table 3 and Figure 3).

## Discussion

IBS-D patients share common characteristics of increased intestinal permeability as well as diarrhea induced by that (Gracie and Ford, 2017). Research pointed out that destruction of the intestinal mucosal barrier is the vital pathophysiological reaction under a series of inflammatory conditions, including IBS-D (Hou et al., 2017). When the barrier was impaired, the enhanced permeability came. With the deepening of research and complexity, scholars stated that the intestinal inflammation and mucosal injuries were correlated with the abnormal regulation of AQP1, 3, 8 (Zhao et al., 2016; Pan et al., 2017). Later, the decline of AQP3 in the intestinal mucosa reinforced the key role of AQPs in the pathophysiological mechanism of diarrhea in IBS-D patients (Camilleri et al., 2019). The decreased AQP1 and AQP3 in our study also confirmed this. It means changes of AQP1 and AQP3 may be essential factors in the pathogenesis and symptom formation of patients with IBS-D. On biopsies from human colon, AQPs were mainly expressed in mucosal epithelial cells. AQP1 was distributed in submucosa and lamina propria of lymphatic endothelial cells, and smooth muscle layer of capillary endothelial cells in the gastrointestinal tract (Nielsen et al., 1993). It exists in lactose endothelial cells in the center of villi of small intestine and produces chyle when digesting food. On the basis of this, an experimental study of AQP1 deficient mice was carried out to verify the impaired fat absorption (Ma et al., 2001), and application of AQP3 inhibitors showed that the decreased protein led to diarrhea as well (Ikarashi et al., 2019). Therefore, we deduce that the declined AQP1 and AQP3 in the intestinal mucosa with IBS-D may cause mucosal injuries, break the intestinal barrier, enhance the permeability and reduce fat absorption, thus leading to diarrhea and other symptoms.

Effects of LncRNA on gene expression during disease development are based on a variety of mechanisms, including the recruitment of chromatin modifiers, controlled recruitment of TFs (activator and inhibitor of gene transcription), regulation of RNA splicing and chromosome loop, dominance of mRNA translation and decay, and the miRNA sponge (Finotti et al., 2019). Found in large amounts of embryonic tissues, LncRNA H19 is silent after birth, serving as an imprinted and maternal transcript (Bartolomei et al., 1991). It has been widely studied in oncology already, and nowadays growing attention has been paid to the role of LncRNA H19 in intestinal inflammation and intestinal barrier gradually. Zou et al. (2016) reported that overexpression of LncRNA increased the abundance of MiR-675p and decreased the mRNA levels of ZO-1 protein and E-cad, thus destroying the integrity of epithelial barrier, and this process could be blocked by the RNA binding protein HuR. In the study of ulcerative colitis, Chen et al. (2016) discovered that LncRNA H19 increased the abundance of targeted vitamin D receptor (VDR) mRNA and decreased the levels of ZO-1 and E-cad mRNA, resulting in intestinal barrier dysfunction. Hence, there might be a close connection between LncRNA H19 and the permeability of intestinal mucosa induced by barrier dysfunction. Though pathogenesis of IBS-D involving the intestinal mucosal barrier has long been affirmed, there is no relevant study on LncRNA H19 so far, we speculate that LncRNA H19 may work in the pathogenesis of IBS-D. As expected, findings in our research authenticated this hypothesis. Expression of LncRNA H19 in the intestinal mucosa of IBS-D patients was significantly decreased, showing no difference with the ending of a recent study, in which the down-regulated LncRNA H19 resulted in the inflammatory factor secretion, becoming a molecular change peculiar to barrier dysfunction, and there was a significant positive relationship between H19 and AQP1 expression level (Fang et al., 2018). Since IBS-D is accompanied by dysregulated inflammatory responses, and for the important role of H19 in inflammatory responses, the sponge in IBS-D should be concerned. As reported, the level of miR-874 was correlated with the expression of LncRNA H19 and AQP3, indicating that LncRNA H19, as a competing endogenous RNA (ceRNA) of miR-874, regulates AQP3 expression through the sponge, leading to intestinal barrier dysfunction (Su et al., 2016). Zhi also stated miR-874 promoted intestinal barrier dysfunction through targeting AQP3 (Zhi et al., 2014). Other scholars held that it’s the activation of autophagy and increased expression of LncRNA H19 in the impaired intestinal mucosa epithelial cells that enhanced the secretion of EGF promoted the repair of intestinal mucosa (Li et al., 2020). All in all, it is reasonable to presume that the decreased expression of LncRNA H19 in the intestinal mucosa of IBS-D patients is related to intestinal barrier dysfunction.

This study was performed on clinical samples and *in vitro* Caco-2 cell models to investigate the expression and effect of H19 on IBS-D prevention. We demonstrated H19 decreased in patients with IBS-D and accompanied by down-regulated AQP1, 3 expression. Similar results were further detected in Caco-2 cells *in vitro*: overexpressed H19 induced increased AQP1 and AQP3, while inhibited H19 expression led to decreased AQP1, 3 expression. So we infer that there should be an intense relationship between H19 and AQP in IBS modulation.

At present, the specific pathogenesis of LncRNA H19 and AQPs in IBS-D cannot be fully elucidated. In terms of the intestinal barrier, insufficient evidence is provided on the complex regulatory mechanisms of LncRNAs. Limitations still exist in present studies, including the lack of *in vivo* and *in vitro* exploration of related mechanism, the signaling pathways related to H19 induced intestinal dysfunction and expression of AQP1, 3. Whether miR-874, autophagy or other secretory factors are the key points in the signaling pathways remain to be studied. Multicentre large sample studies and more specific mechanism pathways are desired to be established and proposed. Even so, the data in this present study could demonstrate the protective effect of LncRNA H19, and AQP1, 3 on intestinal dysfunction with IBS-D. That could be considered as a breakthrough in the study of IBS-D mechanisms, regulating the pathway may be an effective new target for the treatment of IBS.

## Conclusion

In this study, the expressions of LncRNA H19, AQP1, and AQP3 in colonic tissue of IBS-D patients were decreased, and the level of LncRNA was positively correlated with AQP1 and AQP3, suggesting that LncRNA H19 may be involved in regulating the expression of AQP1 and AQP3 in the intestinal mucosa, which may lead to the change of intestinal barrier function in IBS-D patients. LncRNA H19 related regulatory pathways may be a breakthrough in the mechanism of IBS-D, it promises to be a new target for the treatment of IBS-D.

## Data Availability Statement

The datasets used and/or analyzed during the current study are available from the corresponding author on reasonable request.

## Ethics Statement

This study has been approved by the Ethics Committee of Zhejiang Chinese Medical University. The patients/participants provided their written informed consent to participate in this study.

## Author Contributions

GC wrote the manuscript. YY did the experiment. ZW revised the manuscript. SZ guided the manuscript. All authors contributed to the article and approved the submitted version.

## Conflict of Interest

The authors declare that the research was conducted in the absence of any commercial or financial relationships that could be construed as a potential conflict of interest.

## References

[B1] Bartolomei M. S. Zemel S. Tilghman S. M. (1991). Parental imprinting of the mouse H19 gene. *Nature* 351 153–155. 10.1038/351153a0 1709450

[B2] Camilleri M. Carlson P. Chedid V. Vijayvargiya P. Burton D. Busciglio I. (2019). Aquaporin Expression in Colonic Mucosal Biopsies From Irritable Bowel Syndrome With Diarrhea. *Clin. Transl. Gastroenterol.* 10:e00019. 10.14309/ctg.0000000000000019 31033595 PMC6602785

[B3] Chao G. Q. Zhang S. (2018). Aquaporins 1, 3 and 8 expression and cytokines in irritable bowel syndrome rats’colon via cAMP-PKA pathway. *Int. J. Clin. Exp. Pathol.* 11 4117–4123. 31949803 PMC6962796

[B4] Chen S. W. Wang P. Y. Liu Y. C. Sun L. Zhu J. Zuo S. (2016). Effect of Long Noncoding RNA H19 Overexpression on Intestinal Barrier Function and Its Potential Role in the Pathogenesis of Ulcerative Colitis. *Inflamm. Bowel Dis.* 22 2582–2592. 10.1097/mib.0000000000000932 27661667

[B5] Drossman D. A. Hasler W. L. (2016). Rome IV-Functional GI Disorders: Disorders of Gut-Brain Interaction. *Gastroenterology.* 150 1257–1261. 10.1053/j.gastro.2016.03.035 27147121

[B6] Fang Y. Hu J. Wang Z. Zong H. Zhang L. Zhang R. (2018). LncRNA H19 functions as an Aquaporin 1 competitive endogenous RNA to regulate microRNA-874 expression in LPS sepsis. *Biomed. Pharmacother.* 105 1183–1191. 10.1016/j.biopha.2018.06.007 30021355

[B7] Finotti A. Fabbri E. Lampronti I. Gasparello J. Borgatti M. Gambari R. (2019). MicroRNAs and Long Non-coding RNAs in Genetic Diseases. *Mol. Diagn. Ther.* 23 155–171. 10.1007/s40291-018-0380-6 30610665 PMC6469593

[B8] Geng H. Bu H. F. Liu F. Wu L. Pfeifer K. Chou P. M. (2018). In Inflamed Intestinal Tissues and Epithelial Cells, Interleukin 22 Signaling Increases Expression of H19 Long Noncoding RNA, Which Promotes Mucosal Regeneration. *Gastroenterology* 155 144–155. 10.1053/j.gastro.2018.03.058 29621481 PMC6475625

[B9] Gracie D. J. Ford A. C. (2017). Irritable Bowel Syndrome-Type Symptoms Are Associated With Psychological Comorbidity, Reduced Quality of Life, and Health Care Use in Patients With Inflammatory Bowel Disease. *Gastroenterology* 153 324–325. 10.1053/j.gastro.2017.05.037 28572008

[B10] Hou Q. Huang Y. Zhu S. Li P. Chen X. Hou Z. (2017). MiR-144 Increases Intestinal Permeability in IBS-D Rats by Targeting OCLN and ZO1. *Cell Physiol. Biochem.* 44 2256–2268. 10.1159/000486059 29258088

[B11] Hou Q. Zhu S. Zhang C. Huang Y. Guo Y. Li P. (2019). Berberine improves intestinal epithelial tight junctions by upregulating A20 expression in IBS-D mice. *Biomed. Pharmacother* 118:109206. 10.1016/j.biopha.2019.109206 31306972

[B12] Ikarashi N. Mizukami N. Kon R. Kaneko M. Uchino R. Fujisawa I. (2019). Study of the Mechanism Underlying the Onset of Diabetic Xeroderma Focusing on an Aquaporin-3 in a Streptozotocin-Induced Diabetic Mouse Model. *Int. J. Mol. Sci.* 20:3782. 10.3390/ijms20153782 31382467 PMC6696158

[B13] Juan V. Crain C. Wilson C. (2000). Evidence for evolutionarily conserved secondary structure in the H19 tumor suppressor RNA. *Nucleic Acids Res.* 28 1221–1227. 10.1093/nar/28.5.1221 10666466 PMC102599

[B14] Kesuma Y. Firmansyah A. Bardosono S. Sari I. P. Kurniawan A. (2019). Blastocystis ST-1 is associated with Irritable Bowel Syndrome-diarrhoea (IBS-D) in Indonesian adolescences. *Parasite Epidemiol. Control* 6:e00112. 10.1016/j.parepi.2019.e00112 31528737 PMC6742775

[B15] Lei Y. Guo W. Chen B. Chen L. Gong J. Li W. (2018). Tumor-released lncRNA H19 promotes gefitinib resistance via packaging into exosomes in non-small cell lung cancer. *Oncol Rep.* 40 3438–3446. 30542738 10.3892/or.2018.6762 PMC6196604

[B16] Leighton P. A. Ingram R. S. Eggenschwiler J. Efstratiadis A. Tilghman S. M. (1995). Disruption of imprinting caused by deletion of the H19 gene region in mice. *Nature* 375 34–39. 10.1038/375034a0 7536897

[B17] Li C. Zhuang M. Zhu B. Li Y. Zhang W. Yan H. (2020). Epidermal growth factor regulation by autophagy-mediated lncRNA H19 in murine intestinal tract after severe burn. *J. Cell Mol. Med.* 24 5878–5887. 10.1111/jcmm.15262 32301281 PMC7214185

[B18] Ma T. Jayaraman S. Wang K. S. Song Y. Yang B. Li J. (2001). Defective dietary fat processing in transgenic mice lacking aquaporin-1 water channels. *Am. J. Physiol. Cell. Physiol.* 280 C126–C134. 11121384 10.1152/ajpcell.2001.280.1.C126 PMC3491804

[B19] Nielsen S. Smith B. L. Christensen E. I. Agre P. (1993). Distribution of the aquaporin CHIP in secretory and resorptive epithelia and capillary endothelia. *Proc. Natl. Acad. Sci. USA* 90 7275–7279. 10.1073/pnas.90.15.7275 8346245 PMC47119

[B20] Pan J. X. (2017). LncRNA H19 promotes atherosclerosis by regulating MAPK and NF-kB signaling pathway. *Eur. Rev. Med. Pharmacol. Sci.* 21 322–328. 28165553

[B21] Pan L. Y. Chen Y. F. Li H. C. Bi L. M. Sun W. J. Sun G. F. (2017). Dachengqi Decoction Attenuates Intestinal Vascular Endothelial Injury in Severe Acute Pancreatitis in Vitro and in Vivo. *Cell Physiol. Biochem.* 44 2395–2406. 10.1159/000486155 29262394

[B22] Su Z. Zhi X. Zhang Q. Yang L. Xu H. Xu Z. (2016). LncRNA H19 functions as a competing endogenous RNA to regulate AQP3 expression by sponging miR-874 in the intestinal barrier. *FEBS Lett.* 590 1354–1364. 10.1002/1873-3468.12171 27059301

[B23] Thomas A. A. Biswas S. Feng B. Chen S. Gonder J. Chakrabarti S. (2019). lncRNA H19 prevents endothelial-mesenchymal transition in diabetic retinopathy. *Diabetologia* 62 517–530. 10.1007/s00125-018-4797-6 30612136

[B24] Xiao Z. Qiu Y. Lin Y. Medina R. Zhuang S. Rosenblum J. S. (2019). Blocking lncRNA H19-miR-19a-Id2 axis attenuates hypoxia/ischemia induced neuronal injury. *Aging* 11 3585–3600. 10.18632/aging.101999 31170091 PMC6594804

[B25] Zhang W. Xu Y. Chen Z. Xu Z. Xu H. (2011). Knockdown of aquaporin 3 is involved in intestinal barrier integrity impairment. *FEBS Lett.* 585 3113–3119. 10.1016/j.febslet.2011.08.045 21907710

[B26] Zhao G. X. Dong P. P. Peng R. Li J. Zhang D. Y. Wang J. Y. (2016). Expression, localization and possible functions of aquaporins 3 and 8 in rat digestive system. *Biotech. Histochem.* 91 269–276. 10.3109/10520295.2016.1144079 26983346

[B27] Zhi X. Tao J. Li Z. Jiang B. Feng J. Yang L. (2014). MiR-874 promotes intestinal barrier dysfunction through targeting AQP3 following intestinal ischemic injury. *FEBS Lett.* 588 757–763. 10.1016/j.febslet.2014.01.022 24462679

[B28] Zou T. Jaladanki S. K. Liu L. Xiao L. Chung H. K. Wang J. Y. (2016). H19 Long Noncoding RNA Regulates Intestinal Epithelial Barrier Function via MicroRNA 675 by Interacting with RNA-Binding Protein HuR. *Mol. Cell Biol.* 36 1332–1341. 10.1128/mcb.01030-15 26884465 PMC4836219

